# Influence of different processing methods on anti-trypsin activity in bovine colostrum

**DOI:** 10.3168/jdsc.2024-0678

**Published:** 2024-12-20

**Authors:** Lukas Trzebiatowski, Plamen Georgiev, Kathrin Büttner, Axel Wehrend

**Affiliations:** 1 Veterinary Clinic for Reproductive Medicine and Neonatology, Justus Liebig University, Giessen, Germany, 35392; 2 Department of Obstetrics, Reproduction and Reproductive Disorders, Faculty of Veterinary Medicine, Trakia University, Stara Zagora, Bulgaria, 6000; 3 Unit for Biomathematics and Data Processing, Justus Liebig University, Giessen, Germany, 35392

## Abstract

Colostrum is important for supplying the bovine neonate with nutrients, bioactive substances, and immunoglobulins to acquire passive immunity. Trypsin inhibitory activity represents a general characteristic of the first bovine colostrum. It is assumed that the anti-trypsin activity serves to protect bioactive molecules in the colostrum from being digested by the calf. The objectives of the study were to establish a test to determine anti-trypsin activity and test the hypothesis that freezing, acidification, and heat treatment alter anti-trypsin activity compared with untreated bovine colostrum. A photometric assay was established to determine anti-trypsin activity. The activity was expressed in milligrams of inhibited trypsin per milliliter of colostrum. Anti-trypsin activity was measured in untreated colostrum, frozen colostrum (−20°C for 24 h), colostrum acidified using 10% formic acid in a way that it was present as 1% in the sample, and colostrum heat treated according to 2 different protocols (60°C for 60 min and 63.5°C for 30 min). In our study, trypsin inhibition in 40 untreated colostrum samples (0.80 mg/mL, SEM 0.03) corresponded to that observed in frozen colostrum samples (0.79 mg/mL, SEM 0.03). In these first analyzed 40 and 59 extra samples (in total 99 samples), the anti-trypsin activity of frozen colostrum (0.85 mg/mL, SEM 0.01) was compared with colostrum subjected to acidification (0.84 mg/mL, SEM 0.01), heat treatment at 60°C for 60 min (0.65 mg/mL, SEM 0.02), and heat treatment at 63.5°C for 30 min (0.61 mg/mL, SEM 0.02). Acidification did not significantly affect trypsin inhibition. Both heat treatment protocols significantly reduced anti-trypsin activity. In the future, when investigating the effects of postharvest storage and processing on bovine colostrum, the influence on anti-trypsin activity should be evaluated in addition to the effects on immunoglobulins and other components.

## Highlights

• We established a test to determine anti-trypsin activity in bovine colostrum.
• We investigated the effects of processing the colostrum on this activity.
• Freezing and thawing, as well as acidification, did not affect the anti-trypsin activity.
• Two different heat treatment protocols both significantly reduced anti-trypsin activity..

## Graphical Abstract

**Figure:**
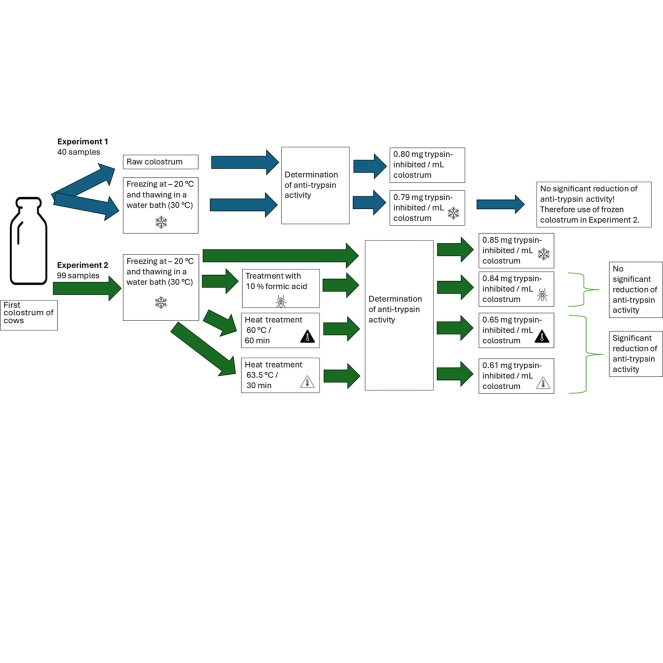
**Summary:** Trypsin-inhibitory activity represents a general characteristic of the first bovine colostrum. The objectives of this study were to establish a test to determine anti-trypsin activity and test the hypothesis that freezing, acidification, and heat treatment would alter anti-trypsin activity compared with untreated bovine colostrum. A photometric assay was established to determine anti-trypsin activity. The activity was expressed in milligrams of inhibited trypsin per milliliter of colostrum. In experiment 1, anti-trypsin activity was compared in untreated and frozen colostrum of the same sample. In 40 untreated colostrum samples, the trypsin-inhibition (0.80 mg/mL) corresponded to that observed in frozen samples (0.79 mg/mL). In experiment 2, we compared the antitrypsin activity of 99 samples of frozen colostrum (0.85 mg/mL) with aliquots subjected to acidification (0.84 mg/mL), heat treatment at 60°C for 60 min (0.65 mg/mL), and heat treatment at 63.5°C for 30 min (0.61 mg/mL). Acidification did not significantly affect trypsin inhibition. Both heat treatment protocols significantly reduced anti-trypsin activity.

Research efforts are being increasingly directed toward examining the impact of providing high-quality colostrum for the calf (Westhoff et al., 2024). In addition to the transfer of immunoglobulins for the development of the immune system in the bovine neonate, colostrum is used for nutrition and also affects future performance (Chuck et al., 2024).

Laskowski and Laskowski (1951) discovered trypsin inhibitory activity as a characteristic of bovine colostrum. Various proteins with trypsin inhibitory activity were isolated from colostrum and characterized (Laskowski and Laskowski, 1951; Pedersen et al., 1971). In these studies, acid stability of certain proteins with anti-trypsin activity was observed. Anti-trypsin activity has been hypothesized to have a protective function for the immunoglobulins in the colostrum by safeguarding them from digestion. This mechanism has a positive influence on the transfer of immunoglobulins (Baintner, 2007; Trzebiatowski et al., 2022). With the advances in molecular methods, the gene encoding for a colostrum-specific bovine trypsin inhibitor (**CTI**) was identified in the bovine genome, which is related to the gene encoding for early lactation protein of marsupials. Proteins related to CTI have been identified in carnivores and artiodactyls (Pharo et al., 2012). In humans and horses, this gene is only present as a pseudogene (Pharo et al., 2016). In a previous study using proteomics methods, 208 proteins were identified and quantified in bovine colostrum. Approximately 10% of these proteins were assigned to the group of protease inhibitors (Zhang et al., 2015). A more precise differentiation of the proteins with trypsin inhibitory activity was not carried out in this study. Proteins with trypsin inhibitory activity decrease significantly in quantity during the transition from colostrum to mature milk (Honkenen-Buzalski and Sandholm, 1981; Yang et al., 2020).

Various processing methods have been described for preserving or reducing the microbial load of colostrum (Elizondo-Salazar et al., 2010; Smith et al., 2022). In most studies evaluating these treatments, the effects on colostral immunoglobulins were primarily recorded (Godden et al., 2019). The effect of postharvest storage and different processing methods on many other colostrum components remains unknown.

Heat treatment is a method of processing colostrum. It is used to reduce the microbiological load of colostrum, for example to sanitize cattle herds with paratuberculosis to break the chain of infection via the colostrum to the newborn calf (Donahue et al., 2012; Mann et al., 2020). However, Godden et al. (2015) could not show any difference in the prevalence of *Mycobacterium avium* ssp. *paratuberculosis*-positive animals between a control group and a group fed heat-treated colostrum. A meta-analysis showed that heat treatment protocols above 60°C have a significant negative influence on the absolute IgG content of the colostrum. Protocols ≤60°C for up to 120 min showed an improvement of the serum IgG concentration in calves, whereas protocols between 60°C and 63.5°C showed a significant reduction in serum IgG (Rabaza et al., 2023). With regard to other colostrum components, a previous study has examined the effect of heat treatment on colostral leukocytes, microRNA, and the complement system (Chandler et al., 2023). Leukocytes were no longer functional after freezing and heat treatment. The function of the complement system and microRNAs is reduced in potency after heat treatment compared with untreated colostrum. Furthermore, the bioavailability of iron for the calf can decrease as a result of heat treatment (Ganz et al., 2021).

Another way of processing colostrum to extend storage time is deep freezing. A single freezing and thawing cycle does not negatively affect the immunoglobulin content (Morrill et al., 2015). A third processing option is the acidification of colostrum with formic acid (Collings et al., 2011). Acidification can significantly reduce the bacterial load of the colostrum without reducing the colostral IgG concentration or the ability of the immunoglobulins to neutralize viruses. Furthermore, it had no negative effect on the immunoglobulin transfer to calves (Smith et al., 2022).

To date, few studies have focused on the effect of postharvest storage and processing of the colostrum on anti-trypsin activity. The aim of the present study was to test the hypothesis that deep freezing, acidification, and heat treatment alter anti-trypsin activity compared with that in untreated colostrum. Two experiments were carried out to examine the following questions:

(1) Does freezing and thawing influence the anti-trypsin activity in colostrum compared with that in the untreated colostrum?
(2) What influence does acidification with formic acid and 2 different heat treatment protocols have on the anti-trypsin activity in bovine colostrum?

The colostrum was harvested from cows of different breeds and parities from the patient population at the Veterinary Clinic for Reproductive Medicine and Neonatology of Justus Liebig University Giessen (Giessen, Germany) as part of the routine udder examination postpartum between October 2021 and February 2022. The breeds included were 68 German Holstein, 17 Simmental, 12 Charolais, and 2 others. The parities of the cows included were 32 first parity, 32 second parity, 18 third parity, 14 fourth parity, and 3 fifth parity. The use of residual colostrum after this examination was authorized by the competent authority (file number kTV8-2017, Regierungspräsidium Giessen, Giessen, Germany).

Within 30 min after calving, the whole quantity of colostrum was harvested, and a composite sample of 100 mL out of all 4 quarters was used for further investigation. The colostrum was collected after cleaning the teat and disinfecting the teat tip.

For the first experiment, each of the colostrum samples (n = 40) was divided into 2 sterile sample containers (Sarstedt, Nümbrecht, Germany). One aliquot (hereafter referred to as “untreated”) was refrigerated at 4°C for a maximum of 8 h after collection, followed by the determination of the anti-trypsin activity. The other aliquot (hereafter referred to as frozen) was cryopreserved at −20°C for 24 h and thawed in a water bath (GFL, Burgwedel, Germany) at 30°C until the sample reached a temperature of 30°C. After thawing, the sample was mixed well by carefully shaking before the anti-trypsin activity was determined.

In experiment 2, the samples were harvested in the same way as in experiment 1. As experiment 1 showed no significant difference in anti-trypsin activity between untreated and frozen colostrum, the second experiment was carried out using frozen colostrum. For experiment 2, the colostrum samples (n = 99) were aliquoted to 3 sterile sample containers (Sarstedt, Nümbrecht, Germany). A schematic of the procedure for experiment 2 can be found in Figure 1.

**Figure 1:**
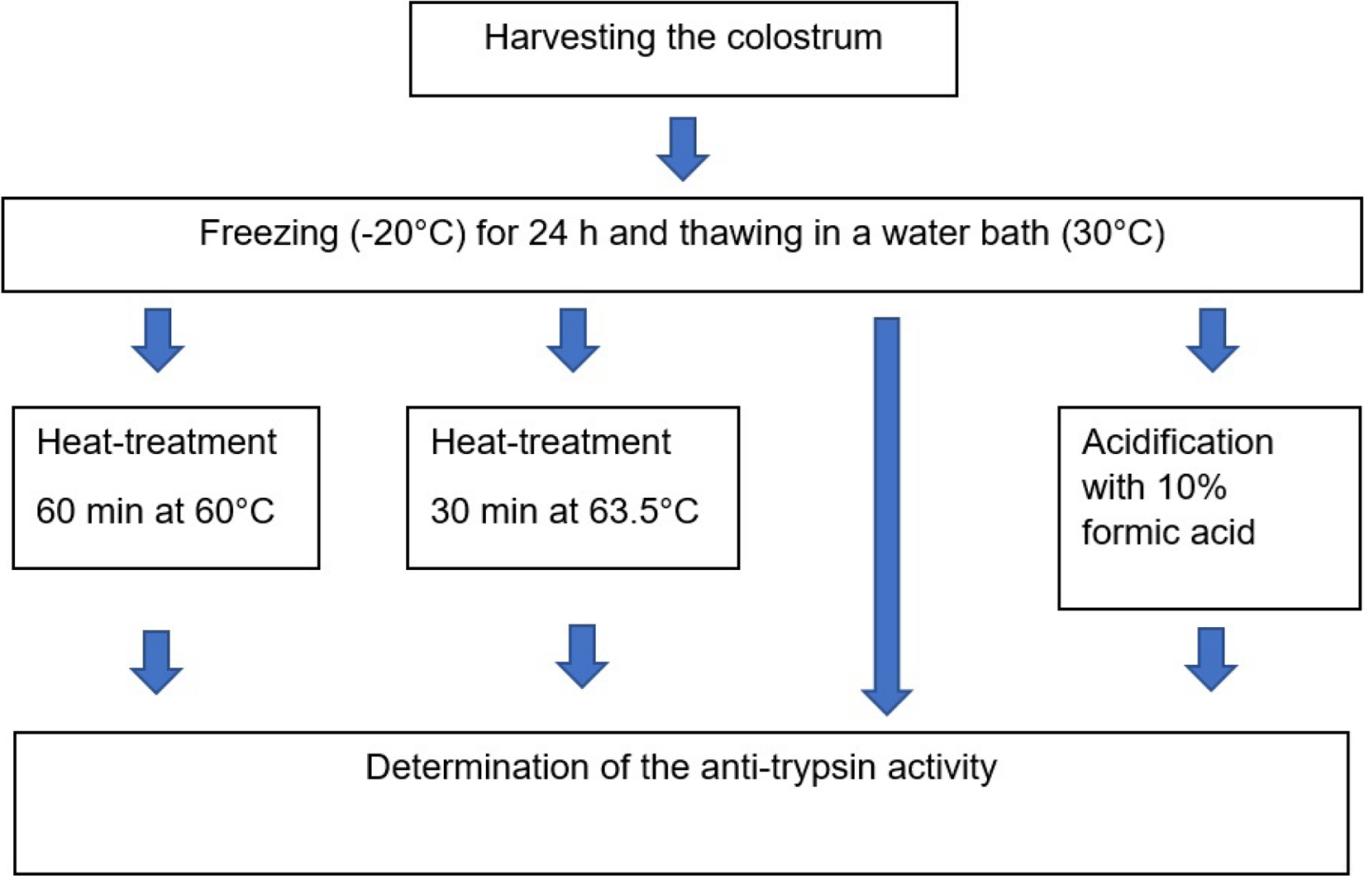
Schematic of the processing of the colostrum samples in experiment 2.

All aliquots were cryopreserved at −20°C for 24 h and thawed in a water bath (GFL, Burgwedel, Germany) at 30°C until the samples reached a temperature of 30°C. After thawing, each sample was mixed well by carefully shaking before further processing or determination of anti-trypsin activity.

Two different heat treatment protocols were used. One aliquot of the samples was heated in a water bath (GFL, Burgwedel, Germany) to 63.5°C for 30 min. As a second heat treatment protocol, another aliquot of the samples was heated at 60°C for 60 min in a water bath (GFL, Burgwedel, Germany). The third aliquot was acidified with 10% formic acid. The formic acid was prepared by diluting 20% formic acid (Roth, Karlsruhe, Germany) with distilled water. The formic acid was added in such a way that it was present as 1% in the colostrum mixture.

For the determination of anti-trypsin activity, the method described by Erlanger et al. (1961) was modified. The measurement is based on the principle of inhibition of the enzymatic conversion of *N*-benzoyl-d,l-arginine-p-nitroanilide by trypsin to p-nitroaniline and is carried out using a photometer. The colorimetric assay kit for trypsin activity (order number MAK290) obtained from Sigma-Aldrich (St. Louis, MO) was used to determine the anti-trypsin activity.

Trypsin from the bovine pancreas (Sigma-Aldrich, St. Louis, MO; order number T1426) was used in this study. Trypsin was dissolved in the buffer from the test kit and used at a concentration of 0.00012 g/mL for the measurement.

In the first step, 1 mL of colostrum was centrifuged in an Eppendorf tube with a capacity of 1.5 mL for 10 min at a relative centrifugal force of 3,000 × *g* at 20°C. A fat-rich fraction formed a floating layer on the surface of the sample. The fat layer was removed. The rest of the sample (whey) was mixed by shaking, and 10 µL of the whey was diluted with 40 µL of a buffer from the test kit and centrifuged again for 10 min at a relative centrifugal force of 3,000 × *g* at 20°C. This step produced a clear supernatant, which was removed with a pipette. In the next step, the supernatant was transferred to a new Eppendorf tube, mixed by shaking, and used to determine the trypsin inhibition.

For the test, 40 µL of buffer, 5 µL of colostrum whey, 5 µL of the trypsin solution, and 50 µL of the reaction mixture were added to a 96-well plate (Nunc, Roskilde, Denmark; order number NAL-469949) for each preparation. A duplicate preparation was performed for each sample. The intrasample CV was 8%.

The measurement was performed in a kinetic measurement procedure photometrically at 405 nm over 20 min with a measurement interval of one min using the Dynatech MRX microplate reader photometer (Dynatech Laboratories, Schaffhausen, Switzerland) and the Revelation software (version 4.25, Dynex Technologies, Denkendorf, Germany). The activity was expressed in milligrams of inhibited trypsin per milliliter of colostrum.

The test was established in advance of the experiments. Following the recommendations of the International Council for Standardization in Haematology, the precision was determined within a series and across different series (Briggs et al., 2014). To determine the precision within a series in 1 sample with high (1.40), medium (1.0), and low activity (0.4), a single run of 10 measurements was performed. The SEM was 0.03 (high), 0.02 (medium), and 0.01 (low). The CV was 8% (high), 6% (medium), and 4% (low).

To determine the precision over different series, the samples were preserved by deep freezing at −20°C and evaluated daily over 5 d. A duplicate preparation was performed for each sample. The SEM was 0.04 (high), 0.02 (medium), and 0.01 (low). The coefficients of variation were 10% (high), 7% (medium), and 10% (low).

The statistical analysis was performed using SAS 9.4 (Statistical Analysis System Institute Inc., Cary, NC).

In experiment 1, an ANOVA with repeated measures was carried out with activity as the dependent variable and processing as the fixed effect. As the differences of the 2 datasets (untreated and frozen) did not show a normal distribution, a pairwise comparison was carried out using the Wilcoxon signed-rank test.

To determine whether there was a difference between the 4 processing methods (frozen, acidified, heat treated for 60 min at 60°C, and heat treated for 30 min at 63.5°C) in experiment 2, an ANOVA with repeated measures with the dependent variable activity and the fixed effect processing was carried out. A Friedman test was performed for the global *P*-value. The multiple pairwise comparisons using the Wilcoxon signed-rank test were adjusted with the Bonferroni correction. In both experiments, statistical significance was declared at *P* < 0.05.

In experiment 1, we compared the trypsin inhibition (in milligrams of inhibited trypsin per milliliter of colostrum) between 40 aliquots of untreated and frozen colostrum. The results are shown in Table 1. No significant difference was found between untreated and frozen colostrum.

**Table 1:** Comparison of anti-trypsin activity (mg of inhibited trypsin per mL of colostrum) between untreated and frozen colostrum

Processing	N	Mean	SEM	Minimum	Maximum	Lower quartile	Median	Upper quartile	*P*-value
Untreated	40	0.80	0.03	0.26	0.99	0.73	0.82	0.96	—
Frozen	40	0.79	0.03	0.26	0.97	0.70	0.81	0.95	1.0

In experiment 2, we performed measurements using 99 colostrum samples and determined the anti-trypsin activity (in milligrams of inhibited trypsin per milliliter of colostrum) from colostrum that was frozen, acidified, heat treated at 60°C for 60 min, and heat treated at 63.5°C for 30 min. The results are presented in Table 2.

**Table 2:** Comparison of anti-trypsin activity (mg of inhibited trypsin per mL of colostrum) in colostrum processed by different methods

Processing	N	Mean	SEM	Minimum	Maximum	Lower quartile	Median	Upper quartile	*P*-values between different treatments
Frozen	99	0.85	0.01	0.57	1.00	0.77	0.87	0.96	Acidified: *P* = 1.00 60°C for 60 min: *P* < 0.001 63.5°C for 30 min: *P* < 0.01
Acidified	99	0.84	0.01	0.29	1.00	0.77	0.86	0.96	Frozen: *P* = 1.00 60°C for 60 min: *P* < 0.01 63.5°C for 30 min: *P* < 0.01
60°C for 60 min	99	0.65	0.02	0.16	0.99	0.59	0.68	0.80	Frozen: *P* < 0.01 Acidified: *P* < 0.01 63.5°C for 30 min: *P* = 0.27
63.5°C for 30 min	99	0.61	0.02	0.17	0.96	0.36	0.65	0.78	Frozen: *P* < 0.01 Acidified: *P* < 0.01 60°C for 60 min: *P* = 0.27

The Friedman test showed a difference in the activity between the different treatments (*P* < 0.001). We did not observe a decrease in anti-trypsin activity after acidification compared with frozen colostrum. Colostrum heat treated for 60 min at 60°C as well as heat treated for 30 min at 63.5°C decreased anti-trypsin activity compared with frozen or acidified colostrum. All these pairwise comparisons were significant. No significant difference was found between the 2 heat treatment protocols.

Anti-trypsin activity in colostrum is seen as a characteristic that facilitates the passage of antibodies through the gastrointestinal tract. Although anti-trypsin activity was after the first description initially intensively investigated, few recent studies have investigated this topic.

In our study, the anti-trypsin activities in untreated colostrum, with an average of 0.8 mg of inhibited trypsin per milliliter of colostrum, correspond to those reported by Piñeiro et al. (1978). Other authors determined lower values of 0.2 to 0.64 mg of inhibited trypsin per milliliter of colostrum (Laskowski and Laskowski, 1951; Pedersen et al., 1971; Honkenen-Buzalski and Sandholm, 1981; Quigley et al., 1995a; Sroka et al., 1998). All these authors used frozen colostrum or did not specify whether they used untreated or frozen colostrum. A comparison between the activity in untreated and frozen colostrum has not yet been carried out. As no differences could be detected in experiment 1, frozen colostrum was used for comparison in experiment 2. For practical calf feeding, it can therefore be concluded that cryopreservation does not reduce anti-trypsin activity. We did not investigate whether different thawing regimens had an influence.

The protocol chosen for acidifying the colostrum with formic acid was a method known for its lack of adverse events on immunoglobulin content (Smith et al., 2022). Acidification did not influence the anti-trypsin activity in the colostrum in our study. The demonstrated acid stability of anti-trypsin activity is thereby consistent with data from older studies (Laskowski and Laskowski, 1951; Pedersen et al., 1971). This appears to be a typical characteristic of the anti-trypsin activity of bovine colostrum. In contrast, anti-trypsin activity in horses is not acid-stable (Baintner and Csapó, 1996). A possible explanation for the need for acid stability could be the acidic pH values in the abomasum and duodenum of calves (Guilloteau et al., 2009), which the proteins with anti-trypsin activity must survive to protect the immunoglobulins from digestion until they reach the site of absorption. Attempts to improve the transfer of immunoglobulins from colostrum to calves have been made by adding trypsin inhibitors from soybeans with varying results. Quigley et al. (1995b) found an improvement in the IgG status of the calves, whereas in contrast, Santoro et al. (2004) found no difference between the groups with and without supplementation of trypsin inhibitors. The different results regarding an improvement in the immunoglobulin supply of calves after the addition of anti-trypsin inhibitors from soy could be due to the lack of acid stability of these proteins or due to different concentrations of trypsin inhibitors.

The 2 heat treatment protocols involving heating at 60°C for 60 min (Gelsinger and Heinrichs, 2017) and 63.5°C for 30 min (Hassan et al., 2020) and were selected because they had a low negative impact on the immunoglobulin content in colostrum. In both heat treatment protocols, anti-trypsin activities were significantly reduced in our experiments compared with that of the frozen colostrum. A meta-analysis showed that heat treatment at 60°C for 60 min not only had almost no effect on the immunoglobulin content in the colostrum but also increased the serum immunoglobulin content in calves compared with feeding with native colostrum. Heat treatment between 60°C and 63.5°C decreased the immunoglobulin content in the colostrum as well as the serum immunoglobulins compared with those observed upon feeding with untreated colostrum (Rabaza et al., 2023). The reason for this apparent contradiction between the lower anti-trypsin activity and the higher serum IgG levels could be that although thermal treatment results in a reduction in anti-trypsin activity, this is still sufficient to protect the antibodies from digestion. In addition, the reduced bacterial contamination could have a positive effect on the uptake of IgG.

Anti-trypsin activity in bovine colostrum is not affected by freezing and thawing or by acidification with formic acid. Heat treatment can lead to a significant reduction in anti-trypsin activity. The biological significance of these results is still unclear and needs to be analyzed by further research. When investigating the effects of postharvest storage and processing on bovine colostrum, in addition to the effects on immunoglobulins and other components (e.g., leukocytes and complement factors), the influence on anti-trypsin activity should also be evaluated.
